# Risk of Paradoxical Eczema in Patients Receiving Biologics for Psoriasis

**DOI:** 10.1001/jamadermatol.2023.4846

**Published:** 2023-12-06

**Authors:** Ali Al-Janabi, Oras A. Alabas, Zenas Z. N. Yiu, Amy C. Foulkes, Steve Eyre, Adnan R. Khan, Nick J. Reynolds, Catherine H. Smith, Christopher E. M. Griffiths, Richard B. Warren

**Affiliations:** 1 Division of Musculoskeletal and Dermatological Sciences, Faculty of Biology, Medicine and Health, The University of Manchester, Manchester, United Kingdom; 2 Dermatology Centre, Manchester Academic Health Science Centre, Northern Care Alliance NHS Foundation Trust, Manchester, United Kingdom; 3 Centre for Dermatology Research, NIHR Manchester Biomedical Research Centre, University of Manchester, Manchester, United Kingdom; 4 Centre for Genetics and Genomics Versus Arthritis, Centre for Musculoskeletal Research, The University of Manchester, Manchester, United Kingdom; 5 UCB Biopharma, Slough, United Kingdom; 6 Institute of Translational and Clinical Medicine, Royal Victoria Infirmary and NIHR Newcastle Biomedical Research Centre, Department of Dermatology, Medical School, University of Newcastle, Newcastle Hospitals NHS Foundation Trust, Newcastle upon Tyne, United Kingdom; 7 St John’s Institute of Dermatology, School of Basic and Medical Biosciences, Faculty of Life Sciences and Medicine, King’s College London, London, United Kingdom; 8 St John’s Institute of Dermatology, Guy’s and St Thomas’ NHS Foundation Trust, London, United Kingdom

## Abstract

**Importance:**

Biologics used for plaque psoriasis have been reported to be associated with an atopic dermatitis (AD) phenotype, or paradoxical eczema, in some patients. The risk factors for this are unknown.

**Objective:**

To explore risk of paradoxical eczema by biologic class and identify factors associated with paradoxical eczema.

**Design, Setting, and Participants:**

This prospective cohort study used data from the British Association of Dermatologists Biologics and Immunomodulators Register for adults treated with biologics for plaque psoriasis who were seen at multicenter dermatology clinics in the UK and Ireland. Included participants were registered and had 1 or more follow-up visits between September 2007 and December 2022.

**Exposures:**

Duration of exposure to tumor necrosis factor (TNF) inhibitors, interleukin (IL) 17 inhibitors, IL-12/23 inhibitors, or IL-23 inhibitors until paradoxical eczema onset, treatment discontinuation, last follow-up, or death.

**Main Outcomes and Measures:**

Incidence rates of paradoxical eczema, paradoxical eczema risk by biologic class, and the association of demographic and clinical variables with risk of paradoxical eczema were assessed using propensity score–weighted Cox proportional hazards regression models.

**Results:**

Of 56 553 drug exposures considered, 24 997 from 13 699 participants were included. The 24 997 included exposures (median age, 46 years [IQR, 36-55 years]; 57% male) accrued a total exposure time of 81 441 patient-years. A total of 273 exposures (1%) were associated with paradoxical eczema. The adjusted incidence rates were 1.22 per 100 000 person-years for IL-17 inhibitors, 0.94 per 100 000 person-years for TNF inhibitors, 0.80 per 100 000 person-years for IL-12/23 inhibitors, and 0.56 per 100 000 person-years for IL-23 inhibitors. Compared with TNF inhibitors, IL-23 inhibitors were associated with a lower risk of paradoxical eczema (hazard ratio [HR], 0.39; 95% CI, 0.19-0.81), and there was no association of IL-17 inhibitors (HR, 1.03; 95% CI, 0.74-1.42) or IL-12/23 inhibitors (HR, 0.87; 95% CI, 0.66-1.16) with risk of paradoxical eczema. Increasing age (HR, 1.02 per year; 95% CI, 1.01-1.03) and history of AD (HR, 12.40; 95% CI, 6.97-22.06) or hay fever (HR, 3.78; 95% CI, 1.49-9.53) were associated with higher risk of paradoxical eczema. There was a lower risk in males (HR, 0.60; 95% CI, 0.45-0.78).

**Conclusions and Relevance:**

In this study, in biologic-treated patients with psoriasis, paradoxical eczema risk was lowest in patients receiving IL-23 inhibitors. Increasing age, female sex, and history of AD or hay fever were associated with higher risk of paradoxical eczema. The overall incidence of paradoxical eczema was low. Further study is needed to replicate these findings.

## Key Points

**Question:**

What factors are associated with paradoxical eczema occurring in patients with psoriasis treated with biologics?

**Findings:**

In this cohort study of 24 997 biologic exposures in 13 699 patients with psoriasis, risk of paradoxical eczema was lowest in patients receiving interleukin 23 inhibitors compared with other biologic classes. Increasing age, history of atopic dermatitis, and history of hay fever were associated with higher risk of paradoxical eczema; risk was lower in males.

**Meaning:**

The findings suggest that interleukin 23 inhibitors could be considered in patients with psoriasis with factors associated with paradoxical eczema.

## Introduction

While biologics targeting tumor necrosis factor (TNF), interleukin (IL) 12/23, IL-17, and IL-23 are highly effective treatments for plaque psoriasis, they are associated with cutaneous adverse events, such as paradoxical psoriasis,^1^ cutaneous lupus, and granulomatous disorders.^2^ Some patients with psoriasis develop paradoxical eczema, an atopic dermatitis (AD) phenotype, during biologic exposure^3^ because these dermatoses are genetically and immunologically divergent and rarely occur together^4,5,6,7^; 1 meta-analysis identified a 2% prevalence of AD in those with psoriasis.^8^ Furthermore, there have been reports of psoriasis or inflammatory arthritis developing secondary to IL-4/13 inhibitors used for AD.^9,10^

In addition to the direct impact of paradoxical eczema, this could result in treatment discontinuation or use of concomitant immunosuppressants.^3^ It is unclear whether paradoxical eczema risk varies by biologic class or other clinical features. The British Association of Dermatologists Biologics and Immunomodulators Register (BADBIR) has recruited more than 20 000 patients with psoriasis from 168 centers in the UK and Ireland.^11^ We used BADBIR to undertake a prospective cohort study to assess (1) the overall and biologic class–specific incidence of paradoxical eczema, (2) whether risk of paradoxical eczema differs between TNF inhibitors and other biologic classes, and (3) the demographic and clinical factors associated with paradoxical eczema.

## Methods

The Strengthening the Reporting of Observational Studies in Epidemiology (STROBE) reporting guideline was followed for the reporting of this cohort study. This work was completed under the ethics approvals of BADBIR. BADBIR was approved in March 2007 by the National Health Service Research Ethics Committee North West England. Written informed consent was obtained from all participants before enrollment.

### Study Participants

Using BADBIR data (from September 2007 to December 2022), we included adults aged 18 years or older with plaque psoriasis exposed to 1 of the following biologics (biosimilars treated the same as originators): TNF inhibitors (adalimumab, certolizumab pegol, etanercept, or infliximab), IL-17 inhibitors (bimekizumab, brodalumab, ixekizumab, or secukinumab), IL-12/23 inhibitors (ustekinumab), or IL-23 inhibitors (guselkumab, risankizumab, or tildrakizumab). As previously described,^12^ data were collected contemporaneously at baseline, every 6 months for the first 3 years, and annually thereafter. Data collected included systemic therapy start and stop dates, baseline comorbidities, adverse event data, and baseline demographics. Ethnicity was included alongside other variables in this study to explore its association with paradoxical eczema. Ethnicity was classified by study participants. Options, defined on the baseline registration questionnaire, included Black, Chinese, South Asian, White, and other (with free text for participants to specify other ethnicities). Exposures with only 1 recorded entry and no follow-up were excluded (Figure).

**Figure.**
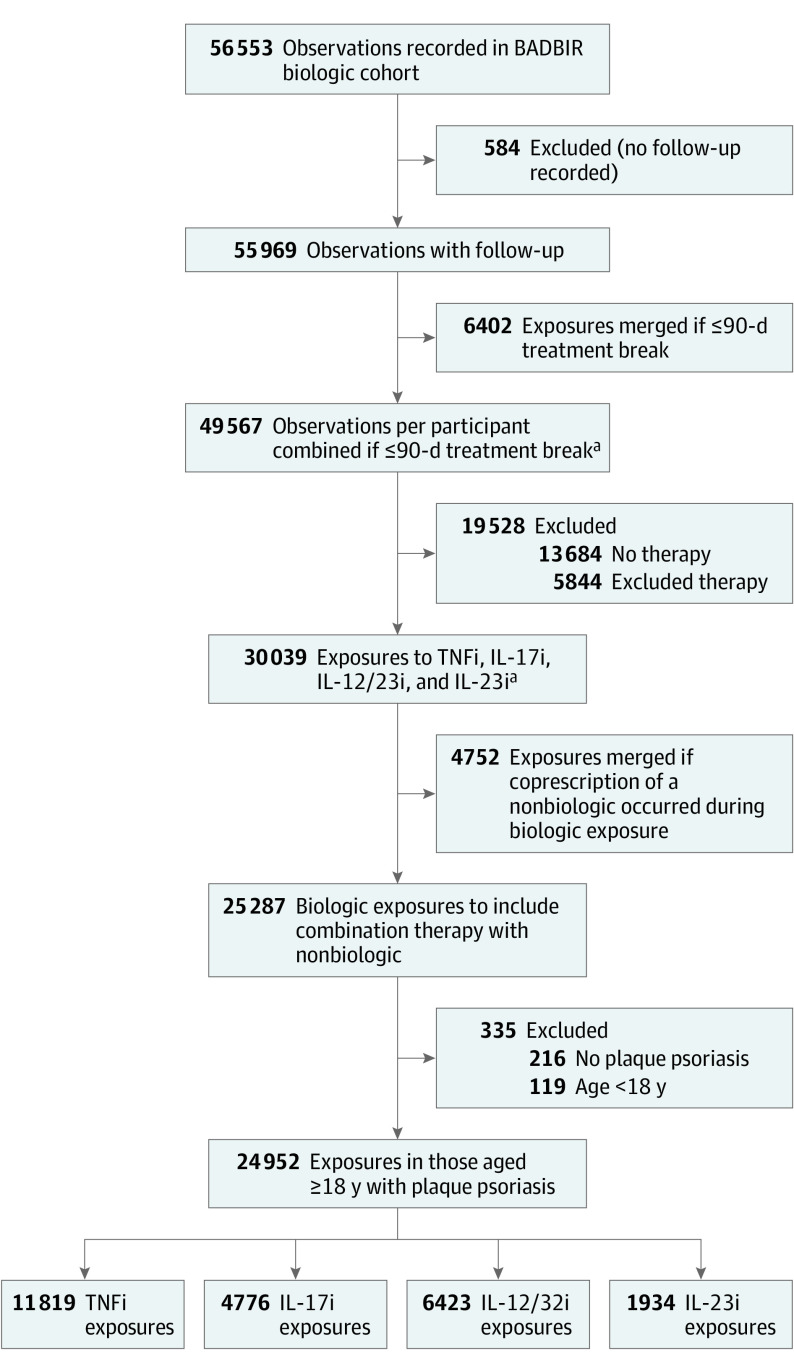
Flow Diagram of Exposure Numbers and Exclusion Reasons BADBIR indicates British Association of Dermatologists Biologics and Immunomodulators Register; IL-12/23i, interleukin 12/23 inhibitor; IL-17i, interleukin 17 inhibitor; IL-23i, interleukin 23 inhibitor; and TNFi, tumor necrosis factor inhibitor. ^a^Merged only if exposures were of the same drug or drug combination. Biosimilar and originator drugs were considered the same.

### Cohort Study Design

For the primary analysis, all lines of exposure were included. Exposures contributed follow-up time until 1 of the following occurrences: (1) paradoxical eczema onset, (2) treatment switch or discontinuation, (3) last documented follow-up, or (4) death. An exposure was considered continuous if there was a treatment break of 90 days or less before restarting the same biologic. Exposures with concomitant conventional systemic or small-molecule therapies were included. A risk window of 90 days was used for the primary analysis; if a paradoxical eczema event occurred within 90 days of switching to another biologic, it was counted as an event for both biologic agents. The final sample size was determined by the number of eligible exposures.

### Paradoxical Eczema Event Definition

Paradoxical eczema events were identified by reviewing adverse event data, including clinical descriptions from the participant’s case records. Events containing the terms *eczema*, *eczematized*, *eczematous*, *atopy*, *atopic*, or *dermatitis* were screened. Potential events were reviewed by 2 researchers (A.A.-J. and R.B.W.) and included if they were described as eczema, atopic eczema, atopic dermatitis, or psoriasiform eczema or eczematized psoriasis. Events were excluded if they were noneczema events (eg, perioral dermatitis), alternative phenotypes (eg, contact dermatitis), or duplicate records.

### Statistical Analyses

Statistical analyses were undertaken in Stata, version 14.2 (StataCorp LLC).^13^ Crude incidence rates of paradoxical eczema and descriptive statistics for paradoxical eczema events (the eMethods in Supplement 1 give more detail) are provided. For the primary analysis, we used a Cox proportional hazards regression survival analysis model to compare the probability of paradoxical eczema in those receiving IL-17 inhibitors, IL-12/23 inhibitors, or IL-23 inhibitors compared with TNF inhibitors.^14^ This was repeated for individual biologics vs adalimumab. Because all lines of exposure were included, we used the vce(cluster id) command in Stata to adjust for the correlation of observations coming from the same participant. We tested for the validity of the proportional hazards assumption for Cox modeling using Schoenfeld residuals.

To adjust for confounders, we generated biologic class propensity scores for inverse-probability treatment weighting for each observation.^15,16^ After weighting, the covariate balance between treatment arms was equivalent (eTable 1 in Supplement 1). To maximize precision of the effect estimate and minimize bias, the propensity score model included covariates suspected to be associated with the outcome but not the exposure as well as potential confounders (the eMethods in Supplement 1 gives for more detail on confounder selection).^17^

For our primary analysis, missing data for included covariates was 0.21% or less (eTable 2 in Supplement 1). The 20 participants with missing ethnicity data were allocated to the other category. Participants with missing data on previous alternative psoriasis phenotypes were presumed not to have had the phenotype. For the first-line exposure sensitivity analysis, due to the proportion of missing data in the smoking and alcohol fields (eTable 3 in Supplement 1), missing data were handled with multiple imputation of 20 data sets.^18^ Propensity scores were calculated separately in each data set, and effect estimates were combined using the Rubin rules.^19^

To explore the association between demographic and clinical variables and paradoxical eczema, we repeated the propensity score–weighted survival analysis but included all covariates in 1 model and reported their adjusted coefficients. These variables were the same as the list of potential confounders. To further explore risk of paradoxical eczema by age, we repeated the survival analysis using age categories. We additionally undertook several sensitivity and post hoc analyses (eMethods in Supplement 1). Two-sided *P* < .05 was used for all analyses.

## Results

Of the 56 553 drug exposures considered, the study sample included 24 997 biologic exposures (43% female; 57% male; median age, 46 years; IQR, 36-55 years) from 13 699 adults (aged ≥18 years) with plaque psoriasis recruited to BADBIR (Figure). Among total exposures, ethnicity was Black for 0.6%, Chinese for 0.7%, South Asian for 6%, White for 92%, and other for 2%. Participant characteristics are presented in Table 1. The total exposure time was 81 441 patient-years. Most exposures were to TNF inhibitors (11 855 [47%]) followed by IL-12/23 inhibitors (6430 [26%]), IL-17 inhibitors (4777 [19%]), and IL-23 inhibitors (1935 [8%]). Exposure to 1 or more nonbiologic systemic therapies occurred during 3567 biologic exposures (14%).

**Table 1.** Exposure-Level Characteristics of Included Participants, Stratified by Biologic Class Exposure

Characteristic	Exposures^a^			
	TNFi (n = 11 855)	IL-17i (n = 4777)	IL-12/23i (n = 6430)	IL-23i (n = 1935)
Age, median (IQR), y	45 (36-54)	48 (37-56)	46 (36-55)	49 (38-57)
Sex				
Female	5141 (43)	2086 (44)	2741 (43)	841 (43)
Male	6714 (57)	2691 (56)	3689 (57)	1094 (57)
Ethnicity				
Black	63 (1)	38 (1)	43 (1)	17 (1)
Chinese	80 (1)	39 (1)	45 (1)	13 (1)
South Asian	596 (5)	332 (7)	394 (6)	148 (8)
White	10 852 (92)	4244 (89)	5791 (90)	1712 (88)
Other^b^	264 (2)	124 (3)	157 (2)	45 (2)
Atopy at baseline				
AD	65 (1)	38 (1)	43 (1)	21 (1)
Asthma	1329 (11)	548 (11)	738 (11)	269 (14)
Hay fever	102 (1)	39 (1)	42 (1)	24 (1)
PsA at baseline	3470 (29)	1573 (33)	1395 (22)	417 (22)
Other psoriasis phenotypes				
Erythrodermic	2069 (17)	818 (17)	1149 (18)	320 (16)
Generalized pustular	537 (5)	239 (5)	291 (5)	78 (4)
Palmoplantar pustulosis	270 (2)	107 (2)	123 (2)	43 (2)
Combined with nonbiologic systemic at biologic initiation				
Methotrexate	1179 (10)	399 (8)	445 (7)	115 (6)
Acitretin	115 (1)	43 (1)	72 (1)	15 (1)
Hydroxycarbamide	25 (<1)	12 (<1)	17 (<1)	7 (<1)
Apremilast	22 (<1)	47 (1)	14 (<1)	31 (2)
Mycophenolate mofetil	22 (<1)	5 (<0.1)	13 (<1)	5 (<1)
Cyclosporine	465 (4)	125 (3)	239 (4)	46 (2)
Dimethyl fumarate	72 (1)	10 (<1)	27 (<1)	2 (<1)
PUVA	0	0	1	0
Combined with nonbiologic systemic at any point				
Methotrexate	1956 (16)	601 (13)	747 (12)	147 (8)
Acitretin	252 (2)	75 (2)	153 (2)	22 (1)
Hydroxycarbamide	64 (1)	24 (1)	51 (1)	8 (<1)
Apremilast	40 (<1)	81 (2)	39 (1)	38 (2)
Mycophenolate mofetil	30 (<1)	12 (<1)	20 (<1)	5 (<1)
Cyclosporine	668 (6)	159 (3)	319 (5)	50 (3)
Dimethyl fumarate	102 (1)	18 (<1)	35 (1)	3 (<1)
PUVA	0	0	1	0

Abbreviations: AD, atopic dermatitis; IL-12/23i, interleukin 12/23 inhibitor; IL-17i, interleukin 17 inhibitor; IL-23i, interleukin 23 inhibitor; PsA, psoriatic arthritis; PUVA, psoralen–UV-A; TNFi, tumor necrosis factor inhibitor.

^a^ Data are presented as number (percentage) of exposures unless otherwise indicated.

^b^ Includes ethnicities defined as other by the study participant or missing ethnicity (n = 20).

### Incidence Rates

A total of 265 paradoxical eczema events were attributed to 273 biologic exposures (1% of total); 8 events occurred within the 90-day risk window of a previous biologic exposure. Adjusted incidence rates were 1.22 per 100 000 person-years for IL-17 inhibitors, 0.94 per 100 000 person-years for TNF inhibitors, 0.80 per 100 000 person-years for IL-12/23 inhibitors, and 0.56 per 100 000 person-years for IL-23 inhibitors (Table 2). Drug-specific incidence rates are presented in Table 2.

**Table 2.** Incidence Rates of Paradoxical Eczema by Biologic Class and Drug^a^

Biologic class, drug	Exposures, No.	Total person-time, y	Paradoxical eczema events, No.	Crude incidence rate, per 100 000 person-years (95% CI)	Adjusted incidence rate, per 100 000 person-years (95% CI)
TNFi					
All	11 819	41 027	141	0.92 (0.77-1.08)	0.94 (0.80-1.12)
Adalimumab	8752	32 390	108	0.90 (0.74-1.08)	0.91 (0.76-1.12)
Certolizumab pegol	281	431	3	1.85 (0.60-5.77)	1.98 (0.61-9.99)
Etanercept	2169	6437	24	0.94 (0.62-1.43)	0.96 (0.64-1.52)
Infliximab	617	1769	6	0.93 (0.42-2.08)	0.97 (0.44-2.57)
IL-17i					
All	4776	11 980	53	1.26 (0.97-1.64)	1.22 (0.94-1.61)
Bimekizumab	40	27	0	0	0
Brodalumab	379	744	5	1.87 (0.78-4.51)	1.83 (0.76-5.52)
Ixekizumab	1060	2126	11	1.53 (0.87-2.70)	1.48 (0.85-2.82)
Secukinumab	3297	9084	37	1.15 (0.85-1.58)	1.11 (0.81-1.56)
IL-12/23i					
Ustekinumab	6423	25 150	73	0.78 (0.62-0.99)	0.80 (0.63-1.02)
IL-23i					
All	1934	3428	7	0.72 (0.38-1.39)	0.56 (0.28-1.30)
Guselkumab	1149	2352	3	0.59 (0.25-1.42)	0.37 (0.15-1.26)
Risankizumab	599	832	4	1.31 (0.49-3.48)	1.24 (0.42-5.39)
Tildrakizumab	186	245	0	0	0

Abbreviations: IL-12/23i, interleukin 12/23 inhibitor; IL-17i, interleukin 17 inhibitor; IL-23i, interleukin 23 inhibitor; TNFi, tumor necrosis factor inhibitor.

^a^ All values except crude incidence rates were computed after weighting by propensity scores.

### Descriptive Summary of Paradoxical Eczema Events

The 265 paradoxical eczema events affected 241 participants. The median time to onset from biologic initiation was 294 days (IQR, 120-699 days), with distribution of events skewed toward biologic initiation (eFigure 1 in Supplement 1). Sites commonly affected by eczema included the face and neck (68 [26%]), limbs (61 [23%]), trunk (35 [13%]), and hands or feet (33 [12%]) (eTable 4 in Supplement 1). Pruritus (49 [18%]), redness (18 [7%]), and dryness (11 [4%]) were the most reported symptoms. Of the 21 reported biopsies, all showed spongiosis or a feature of eczema, with 1 having overlapping features of psoriasis. Topical treatments for paradoxical eczema were most common (115 [43%]) followed by oral antibiotics (20 [7%]), stopping or switching biologic therapy (17 [6%]), and systemic corticosteroids (12 [4%]) (eTable 4 in Supplement 1). Of the 241 affected participants, 221 had 1 paradoxical eczema event and 20 had more than 1 event (44 events in total). Of 24 repeated events, 5 (21%) occurred after receipt of the same biologic as for the index event, 6 (25%) after receipt of a different biologic within the same class, and 13 (54%) after receipt of a biologic from another class. Compared with participants with 1 paradoxical eczema event, TNF inhibitors were the most used biologics for the index event in the multiple-event cohort (16 [80%] vs 111 [50%]) followed by IL-17 inhibitors (2 [10%] vs 41 [19%]), IL-12/23 inhibitors (2 [10%] vs 63 [28%]), and IL-23 inhibitors (0 [0%] vs 6 [3%]) (eTable 5 in Supplement 1). A higher proportion of these participants had hay fever (4 [20%] vs 3 [1%]), had psoriatic arthritis (8 [40%] vs 67 [30%]), or received cyclosporine at biologic initiation (5 [25%] vs 18 [8%]) or at any point during biologic therapy (7 [35%] vs 27 [12%]) (eTable 5 in Supplement 1).

### Propensity Score–Weighted Survival Analysis

Compared with TNF inhibitors, IL-23 inhibitors were associated with a lower risk of paradoxical eczema (hazard ratio [HR], 0.39; 95% CI, 0.19-0.81) (Table 3). There was no association of IL-12/23 inhibitors (HR, 0.87; 95% CI, 0.66-1.16) or IL-17 inhibitors (HR, 1.03; 95% CI, 0.74-1.42) with risk of paradoxical eczema. Subgroup analysis (Table 3) identified a lower risk associated with guselkumab compared with adalimumab (HR, 0.28; 95% CI, 0.11-0.71). When guselkumab was used as the reference category, all included biologics except risankizumab were associated with an increased risk of paradoxical eczema (eTable 6 in Supplement 1). On the basis of Schoenfeld residuals, there was no evidence of violation of the proportionality assumption required for the Cox proportional hazards regression model for either biologic classes or individual biologics.

**Table 3.** Propensity Weight–Adjusted Cox Proportional Hazards Regression Survival Models for Risk of Paradoxical Eczema by Biologic Class, Biologic Drug, or Other Covariates

Variable	Hazard ratio (95% CI)	*P* value
Biologic class		
TNFi	1 [Reference]	NA
IL-17i	1.03 (0.74-1.42)	.86
IL-12/23i	0.87 (0.66-1.16)	.35
IL-23i	0.39 (0.19-0.81)	.01
Individual biologics		
Adalimumab	1 [Reference]	NA
Certolizumab pegol	1.35 (0.42-4.32)	.61
Etanercept	0.96 (0.58-1.58)	.87
Infliximab	0.95 (0.42-2.16)	.91
Brodalumab	1.35 (0.55-3.35)	.51
Ixekizumab	1.12 (0.61-2.06)	.72
Secukinumab	0.98 (0.68-1.41)	.90
Bimekizumab^a^	NA	NA
Ustekinumab	0.87 (0.65-1.17)	.35
Guselkumab	0.28 (0.11-0.71)	.008
Risankizumab	0.78 (0.27-2.26)	.65
Tildrakizumab^a^	NA	NA
Baseline demographic variables^b^		
Age^c^	1.02 (1.01-1.03)	.003
Sex		
Female	1 [Reference]	NA
Male	0.60 (0.45-0.78)	<.001
Ethnicity		
Black	1.35 (0.32-5.72)	.68
Chinese	2.81 (1.10-7.18)	.03
South Asian	1.33 (0.70-2.54)	.38
White	1 [Reference]	NA
Other^d^	1.12 (0.49-2.57)	.79
Baseline clinical variables^b^		
Atopic dermatitis	12.40 (6.97-22.06)	<.001
Asthma	0.97 (0.61-1.54)	.90
Hay fever	3.78 (1.49-9.53)	.005
Psoriatic arthritis	1.19 (0.89-1.60)	.24
Erythrodermic psoriasis	1.10 (0.76-1.59)	.60
Generalized pustular psoriasis	0.83 (0.43-1.59)	.58
Palmoplantar pustulosis	1.13 (0.52-2.45)	.75

Abbreviations: IL-12/23i, interleukin 12/23 inhibitor; IL-17i, interleukin 17 inhibitor; IL-23i, interleukin 23 inhibitor; NA, not applicable; TNFi, tumor necrosis factor inhibitor.

^a^ The data for bimekizumab and tildrakizumab are not shown due to unstable effect estimates resulting from the low number of exposures and absence of paradoxical eczema events attributed to these drugs.

^b^ Single-model Cox proportional hazards regression model including all displayed demographic and clinical covariates in addition to biologic class.

^c^ The effect estimates for age represent an increase in hazard ratio per year from the base age of 18 years.

^d^ Includes ethnicities defined as other by the study participant or missing ethnicity (n = 20).

### Association of Demographic and Clinical Covariates With Paradoxical Eczema Risk

When including several baseline variables selected a priori as covariates (Table 3) in a repeated biologic class survival analysis, lower risk of paradoxical eczema was found in men (HR, 0.60; 95% CI, 0.45-0.78). Age (HR, 1.02 per year from age 18 years; 95% CI, 1.01-1.03), prior AD (HR, 12.40; 95% CI, 6.97-22.06), and hay fever (HR, 3.78; 95% CI, 1.49-9.53) were associated with increased risk of paradoxical eczema, but there was no association for asthma (HR, 0.97; 95% CI, 0.61-1.54). There was an apparent increased risk in Chinese participants (HR, 2.81; 95% CI, 1.10-7.18) but no association in other ethnic groups compared with White participants (Table 3). There was no association of other psoriasis phenotypes or psoriatic arthritis with paradoxical eczema. By categorizing age, we identified an increased risk in the 50 to 69 years category (HR, 1.75; 95% CI, 1.02-2.98) and 70 years or older category (HR, 2.52; 95% CI, 1.23-5.20), but there was no association in the 30 to 49 years category (HR, 1.37; 95% CI, 0.81-2.32) compared with those younger than 30 years (eFigure 2 in Supplement 1).

### Sensitivity Analyses

A repeated analysis with a drug-exposure risk window of 0 days demonstrated similar results to the 90-day risk window primary analysis (eTable 7 in Supplement 1). Due to the potential impact of unmeasured time-varying confounders, we repeated the survival analysis restricted to only the first biologic exposure for each participant. This analysis also assessed paradoxical eczema risk by baseline smoking or alcohol consumption status. Of the 11 732 exposures (exposure time, 39 274 patient-years), paradoxical eczema occurred in 141 (1%). The biologic class HRs were similar to those in the primary analysis (eTable 8 in Supplement 1). For individual biologics, a first-line etanercept exposure was associated with lower risk of paradoxical eczema compared with adalimumab (HR, 0.45; 95% CI, 0.22-0.95). The apparent increased risk associated with certolizumab pegol (HR, 5.41; 95% CI, 1.66-17.57) may have been due to a low number of first-line exposures (n = 57) to this drug (eTable 8 in Supplement 1). There was no association with baseline smoking status or alcohol consumption (eTable 8 in Supplement 1).

To evaluate whether our findings were specific to the paradoxical eczema phenotype, we repeated the survival analysis but defined treatment failure as onset of other eczema reactions (n = 156), such as contact dermatitis, seborrheic dermatitis, and stasis dermatitis (eTable 9 in Supplement 1). There were no significant differences in risks for these phenotypes between biologics and biologic classes (eTable 10 in Supplement 1). Although there were no significant differences, the HRs were greater for IL-12/23 inhibitor exposures (HR, 1.07; 95% CI, 0.74-1.56) and IL-23 inhibitor exposures (HR, 1.25; 95% CI, 0.65-2.44) than for TNF inhibitor exposures; the opposite was observed for paradoxical eczema. The direction of the coefficients for age, male sex, AD, and hay fever were the same as for paradoxical eczema, although there was no significant difference for male sex and AD for other eczema phenotypes. Post hoc, we found that the median time to all-cause biologic discontinuation following onset of paradoxical eczema (467 days; IQR, 122-1267 days) was shorter than with other eczema phenotypes (710 days; IQR, 252-1624 days) (*P* = .02) (eFigure 3 in Supplement 1).

To assess the impact of cotreatment with nonbiologic systemic therapies, we repeated the model including this as a binary variable and identified no association with paradoxical eczema (HR, 1.26; 95% CI, 0.89-1.79). When nonbiologic systemics were categorized, we observed an increased risk of paradoxical eczema associated with cyclosporine (HR, 3.28; 95% CI, 2.03-5.30) and no association with methotrexate (HR, 0.62; 95% CI, 0.37-1.04) (eTable 11 in Supplement 1). To account for biologic exposure–related covariates that were not included, such as treatment failure, treatment of previous paradoxical eczema events, or occurrence of other adverse events, we repeated analysis for first-line biologic exposures only. The increased risk of paradoxical eczema associated with concomitant cyclosporine remained (HR, 2.10; 95% CI, 1.12-3.91), and there was no association with methotrexate (HR, 0.78; 95% CI, 0.41-1.47). Of 30 patients receiving cyclosporine at biologic initiation who then developed paradoxical eczema, the recorded onset date of paradoxical eczema was after or on the same day as cyclosporine cessation in 21 cases (median, 0.5 days; IQR, −28 to 101 days), with the distribution demonstrating a left-sided skew (eFigure 4 in Supplement 1), unlike the methotrexate cohort (n = 17; median, −6 days; IQR, −330 to 137 days).

## Discussion

In this study, while the incidence of paradoxical eczema in biologic-treated patients with psoriasis was low overall, it was highest in those receiving IL-17 inhibitors followed by those receiving TNF inhibitors, those receiving IL-12/23 inhibitors, and those receiving IL-23 inhibitors. Compared with TNF inhibitors, IL-23 inhibitor exposure was associated with significantly lower risk of paradoxical eczema; this result may have been attributable mostly to guselkumab due to the low number of exposures to other IL-23 inhibitors in these data. These findings remained when restricting the analysis to first-line biologic exposures and were specific to this eczema phenotype. Increasing age, female sex, prior AD, and prior hay fever were associated with increased risk of paradoxical eczema.

### Interpretation of Findings

To our knowledge, this is the first study to compare paradoxical eczema risk by biologic class. Based on clinical experience and prevalence of eczematous reactions reported in some IL-17 inhibitor clinical trials,^20,21,22,23^ we suspected an association between IL-17 inhibitor exposure and paradoxical eczema. While the incidence of paradoxical eczema was numerically highest among IL-17 inhibitor exposures, it was not significantly different from the incidence among TNF inhibitor exposures. The low overall incidence of paradoxical eczema may be reassuring for patients and clinicians, but it is possible that the incidence was underestimated due to underreporting or exclusion of adverse events with insufficient detail.

The mechanisms of paradoxical eczema are unknown. Some authors have speculated that inhibition of TNF or the IL-17/23 axis permits development of T-helper 2 (Th2)–mediated inflammation, which may otherwise be inhibited by Th1/Th17 activity.^3,24^ Th2-predominant or mixed inflammatory profiles in lesional skin have been described in small case series.^25,26^ The biological basis for IL-23 inhibitors being associated with the lowest risk of paradoxical eczema is unclear.

Regarding subgroup analyses, the reduced risk of paradoxical eczema associated with guselkumab supports the findings of the primary analysis. Etanercept was associated with a lower risk of paradoxical eczema when the analysis was restricted to first-line exposures, possibly because etanercept was more commonly used as a first-line therapy when BADBIR was established and data entry practices have changed or awareness of this adverse event has increased.

As expected and consistent with other studies, we identified an association between paradoxical eczema and previous AD^27,28,29^ and hay fever. This supports an association of genetic factors with atopy, which we previously demonstrated in a genotyped cohort with paradoxical eczema.^30^ The lack of association with asthma could indicate that certain genetic variants more commonly associated with AD and hay fever rather than asthma, such as *FLG* variants,^31^ play an important role in paradoxical eczema.

To our knowledge, the increased risk of paradoxical eczema in females has not been identified previously. While AD is more common in males than in females during childhood, this trend reverses into early adulthood; this finding in our study could reflect this.^32^ We did not observe an increase in paradoxical eczema risk in South Asian participants compared with White participants, but could not conclude on risk in other ethnic groups due to sample size limitations. Unlike a previous study,^27^ we did not identify an increased risk in smokers or those consuming alcohol at baseline.

We were surprised to find an association between cyclosporine use at the time of biologic initiation and paradoxical eczema. Some patients may have had an undocumented active eczema, or an overlapping phenotype, that was unmasked at cyclosporine withdrawal or dose reduction or biologic initiation. Cyclosporine treatment or withdrawal has been previously reported to aggravate or induce relapse in animal autoimmunity models, possibly by suppressing inflammation while allowing antigen-specific priming of T cells, altering Th1/Th2 antagonism,^33^ or inactivating regulatory T cells.^34^

Some patients developed repeated paradoxical eczema events while receiving different biologic classes. This finding may indicate a common immunological mechanism downstream of the drug targets. While there were too few participants for inferential statistical analysis, most patients with multiple events developed their first event while receiving a TNF inhibitor, and a substantial proportion were using cyclosporine at biologic initiation. Assessment of the consequences of specific sequences of therapeutic agents and drug combinations is a challenging but important area for future research.

### Strengths and Limitations

Strengths of this study include the large sample size and inclusion of multiple lines of exposure per participant. We included data for all currently available biologics, originating from more than 160 dermatology centers in the UK and Ireland. We minimized bias from our confounders using propensity score weighting.

The main limitation is the small numbers of observations within certain subgroups, such as specific biologic exposures or participants in ethnic minority groups, restricting generalizability of our findings and the interpretation of some subgroup analyses. The small number of paradoxical eczema events resulted in imprecise effect estimates. The reduced risk observed in association with IL-23 inhibitors should be interpreted with caution, as the number of IL-23 inhibitor exposures was low compared with other classes. There was a risk of adverse event misclassification, as ascertainment of adverse events relied on free-text descriptions derived from medical records, which are subjective. It was also not possible to ascertain whether each paradoxical eczema event was associated with the drug or represented natural occurrence of adult-onset eczema, but the tendency for events to occur close to biologic initiation supports that these were true adverse events.

## Conclusions

In this study, there was a lower risk of paradoxical eczema among participants receiving IL-23 inhibitors. Factors associated with paradoxical eczema included increasing age, female sex, history of AD, and history of hay fever. These findings need replication. Future studies with more exposures and paradoxical eczema events would enable a more robust analysis of individual drugs and patient subgroups.

## Notes

Supplement 1.
eMethods
**eFigure 1.** Days from Biologic Initiation to Onset of Paradoxical Eczema **eFigure 2.** Distribution of Age of Onset in Paradoxical Eczema Cases **eFigure 3.** Days to Biologic Discontinuation Following Onset of Paradoxical Eczema or Other Eczema Phenotypes **eFigure 4****.** Histogram Showing Days From Cessation of Methotrexate or Ciclosporin, Taken Concurrently With a Biologic at Biologic Initiation, to Onset of Paradoxical Eczema **eTable 1.** Confounder Bias Before and After Inverse Probability Treatment Weighting by Propensity Score **eTable 2.** Missing Data From Observations Included in Primary Analysis (n=24,997) **eTable 3.** Missing Data From Observations Included in First-Line Biologic Sensitivity Analysis (n=11,732) **eTable 4.** Features of Paradoxical Eczema Events (n=265) **eTable 5.** Characteristics of Participants With One Versus More Than One Paradoxical Eczema Event (Index Event Only) **eTable 6.** Propensity Weight-Adjusted Cox Proportional Hazards Model of Paradoxical Eczema by Biologic Drug, Using Guselkumab as the Reference Category **eTable 7.** Propensity Weight-Adjusted Cox Proportional Hazards Model of Paradoxical Eczema by Biologic Class or Biologic Drug, Without an Exposure Risk Window (Sensitivity Analysis) **eTable 8.** Propensity Weight-Adjusted Cox Proportional Hazards Model of Paradoxical Eczema by Biologic Class or Biologic Drug, Limited to First-Line Therapy Only (Sensitivity Analysis) **eTable 9.** Number of Exposures Linked to Eczema or Dermatitis Adverse Events Other Than Paradoxical Eczema **eTable 10.** Propensity Weight-Adjusted Cox Proportional Hazards Model for Risk of Other Eczema Phenotypes by Biologic Class, Biologic Drug or Other Covariates (Sensitivity Analysis) **eTable 11.** Propensity Weight-Adjusted Cox Proportional Hazards Model for Risk of Paradoxical Eczema by Biologic Class or Other Covariates, Adjusting Time-Varying Use of Non-Biologic Systemic Therapies (Sensitivity Analysis)
eReferences

Supplement 2.
BADBIR Study Group

Supplement 3.
Data Sharing Statement
