# Supplementary material for: Risk of Paradoxical Eczema in Patients Receiving Biologics for Psoriasis
Source: JAMA Dermatol. 2023 Dec 6;160(1):71–9. doi: 10.1001/jamadermatol.2023.4846 (PMC10701661; doi:10.1001/jamadermatol.2023.4846)
Supplement: Supplement 1. — eMethods eFigure 1. Days from Biologic Initiation to Onset of Paradoxical Eczema eFigure 2. Distribution of Age of Onset in Paradoxical Eczema Cases eFigure 3. Days to Biologic Discontinuation Following Onset of Paradoxical Eczema or Other Eczema Phenotypes eFigure 4. Histogram Showing Days From Cessation of Methotrexate or Ciclosporin, Taken Concurrently With a Biologic at Biologic Initiation, to Onset of Paradoxical Eczema eTable 1. Confounder Bias Before and After Inverse Probability Treatment Weighting by Propensity Score eTable 2. Missing Data From Observations Included in Primary Analysis (n=24,997) eTable 3. Missing Data From Observations Included in First-Line Biologic Sensitivity Analysis (n=11,732) eTable 4. Features of Paradoxical Eczema Events (n=265) eTable 5. Characteristics of Participants With One Versus More Than One Paradoxical Eczema Event (Index Event Only) eTable 6. Propensity Weight-Adjusted Cox Proportional Hazards Model of Paradoxical Eczema by Biologic Drug, Using Guselkumab as the Reference Category eTable 7. Propensity Weight-Adjusted Cox Proportional Hazards Model of Paradoxical Eczema by Biologic Class or Biologic Drug, Without an Exposure Risk Window (Sensitivity Analysis) eTable 8. Propensity Weight-Adjusted Cox Proportional Hazards Model of Paradoxical Eczema by Biologic Class or Biologic Drug, Limited to First-Line Therapy Only (Sensitivity Analysis) eTable 9. Number of Exposures Linked to Eczema or Dermatitis Adverse Events Other Than Paradoxical Eczema eTable 10. Propensity Weight-Adjusted Cox Proportional Hazards Model for Risk of Other Eczema Phenotypes by Biologic Class, Biologic Drug or Other Covariates (Sensitivity Analysis) eTable 11. Propensity Weight-Adjusted Cox Proportional Hazards Model for Risk of Paradoxical Eczema by Biologic Class or Other Covariates, Adjusting Time-Varying Use of Non-Biologic Systemic Therapies (Sensitivity Analysis) eReferences [file jamadermatol-e234846-s001.pdf]

Supplementary Online Content

Al-Janabi A, Alabas OA, Yiu ZZN, et al. Risk of paradoxical eczema in patients with psoriasis treated with biologics. *JAMA Dermatol*. Published online December 6, 2023. doi:10.1001/jamadermatol.2023.4846

eMethods.

eFigure 1. Days from Biologic Initiation to Onset of Paradoxical Eczema.

eFigure 2. Distribution of Age of Onset in Paradoxical Eczema Cases

eFigure 3. Days to Biologic Discontinuation Following Onset of Paradoxical Eczema or Other Eczema Phenotypes

eFigure 4. Histogram Showing Days From Cessation of Methotrexate or Ciclosporin, Taken Concurrently With a Biologic at Biologic Initiation, to Onset of Paradoxical Eczema

eTable 1. Confounder Bias Before and After Inverse Probability Treatment Weighting by Propensity Score

eTable 2. Missing Data From Observations Included in Primary Analysis (n=24,997)

eTable 3. Missing Data From Observations Included in First-Line Biologic Sensitivity Analysis (n=11,732)

eTable 4. Features of Paradoxical Eczema Events (n=265)

eTable 5. Characteristics of Participants With One Versus More Than One Paradoxical Eczema Event (Index Event Only)

eTable 6. Propensity Weight-Adjusted Cox Proportional Hazards Model of Paradoxical Eczema by Biologic Drug, Using Guselkumab as the Reference Category

eTable 7. Propensity Weight-Adjusted Cox Proportional Hazards Model of Paradoxical Eczema by Biologic Class or Biologic Drug, Without an Exposure Risk Window (Sensitivity Analysis)

eTable 8. Propensity Weight-Adjusted Cox Proportional Hazards Model of Paradoxical Eczema by Biologic Class or Biologic Drug, Limited to First-Line Therapy Only (Sensitivity Analysis)

© 2023 Al-Janabi A et al. *JAMA Dermatology.*

eTable 9. Number of Exposures Linked to Eczema or Dermatitis Adverse Events Other Than Paradoxical Eczema

eTable 10. Propensity Weight-Adjusted Cox Proportional Hazards Model for Risk of Other Eczema Phenotypes by Biologic Class, Biologic Drug or Other Covariates (Sensitivity Analysis)

eTable 11. Propensity Weight-Adjusted Cox Proportional Hazards Model for Risk of Paradoxical Eczema by Biologic Class or Other Covariates, Adjusting Time-Varying Use of Non-Biologic Systemic Therapies (Sensitivity Analysis)

References

This supplementary material has been provided by the authors to give readers additional information about their work.

© 2023 Al-Janabi A et al. *JAMA Dermatology.*

eMethods

Descriptive statistics
Additional descriptive statistics for participants who developed multiple paradoxical eczema events were compared numerically with participants who had a single event. For multiple-event statistics, only the first exposure was considered for events associated with more than one exposure due to overlapping risk windows.

Confounder selection
Covariate selection was based on expert opinion and a systematic review of reported cases of paradoxical eczema.[1] These included: age, sex, ethnicity, alcohol consumption, smoking status and history of previous AD, asthma, hay fever, psoriatic arthritis (PsA) or other psoriasis phenotypes (erythrodermic, generalised pustular or palmoplantar pustulosis) at baseline. Age remained a continuous variable during analysis and was adjusted to represent age at the start of each exposure. Comorbidity covariates (atopic diseases, PsA and other psoriasis phenotypes) were recorded prior to initiation of the first-line biologic therapy. Because alcohol consumption and smoking status are time-varying covariates for which data was not available beyond the first-line exposure, we included these in a separate analysis limited to first-line exposures only.

Sensitivity analyses
We undertook several sensitivity analyses to test various assumptions. Firstly, because an adverse event risk period of 90 days is arbitrary and may vary between individual drugs depending on half-life, we repeated the primary analysis using a risk window of 0 days instead of 90 days. Secondly, as described above, we undertook an analysis including only first-line biologic exposures in case our findings could have been influenced by time-varying covariates. Thirdly, to identify whether our findings are specific to the paradoxical eczema phenotype or eczematous reactions in general, we repeated our analysis for those with alternative eczema/dermatitis adverse events, such as contact dermatitis, seborrheic dermatitis and stasis dermatitis. Lastly, we repeated the analysis after adjusting for concomitant non-biologic systemic therapies, because such treatments could confound outcome by either suppressing (or contributing to) the development of an eczema phenotype or be prescribed for it. We included concomitant treatment as a binary variable, indicating whether any of the following were given at the time of biologic initiation: acitretin; apremilast; ciclosporin; dimethyl fumarate; hydroxycarbamide; methotrexate; mycophenolate mofetil. We then repeated this using a categorical variable stratified by drug.

Post hoc analyses
Some of the descriptive statistics relating to paradoxical eczema events, such as treatments used, were derived from free text adverse event descriptions which may not necessarily capture full details of treatment discontinuation related to paradoxical eczema. To further understand the impact of paradoxical eczema on biologic discontinuation, we compared the distributions of days from eczema onset to the end of biologic exposure with the other eczematous phenotypes (Mann-Whitney U test).

© 2023 Al-Janabi A et al. *JAMA Dermatology.*

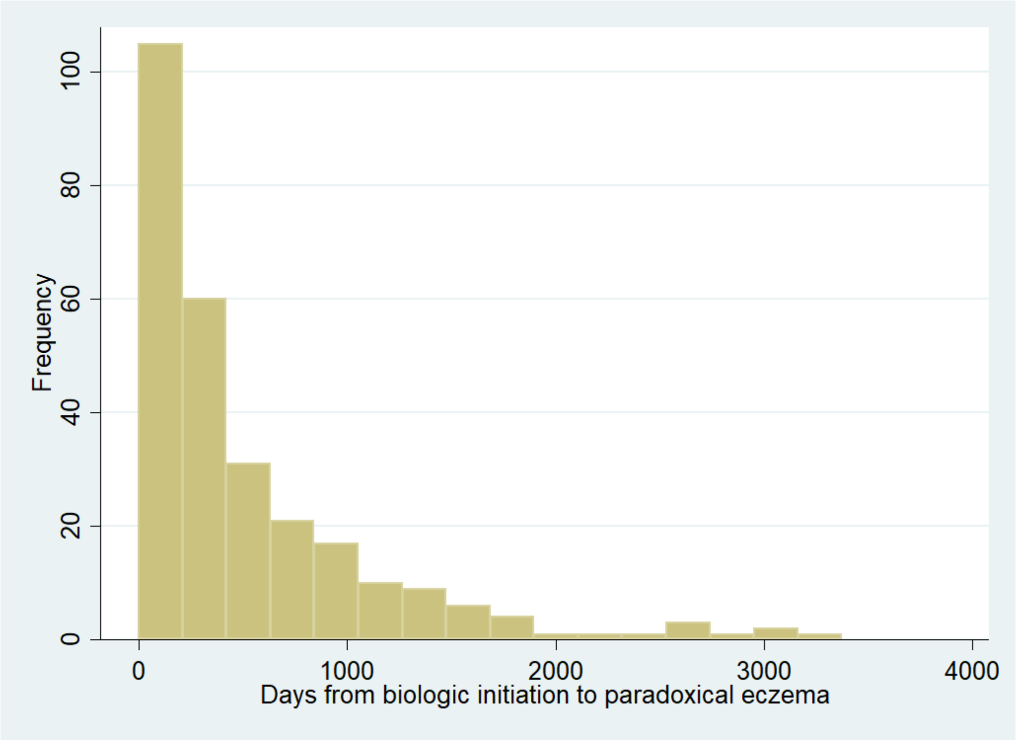

eFigure 1. Days from biologic initiation to onset of paradoxical eczema.

© 2023 Al-Janabi A et al. *JAMA Dermatology.*

A

B

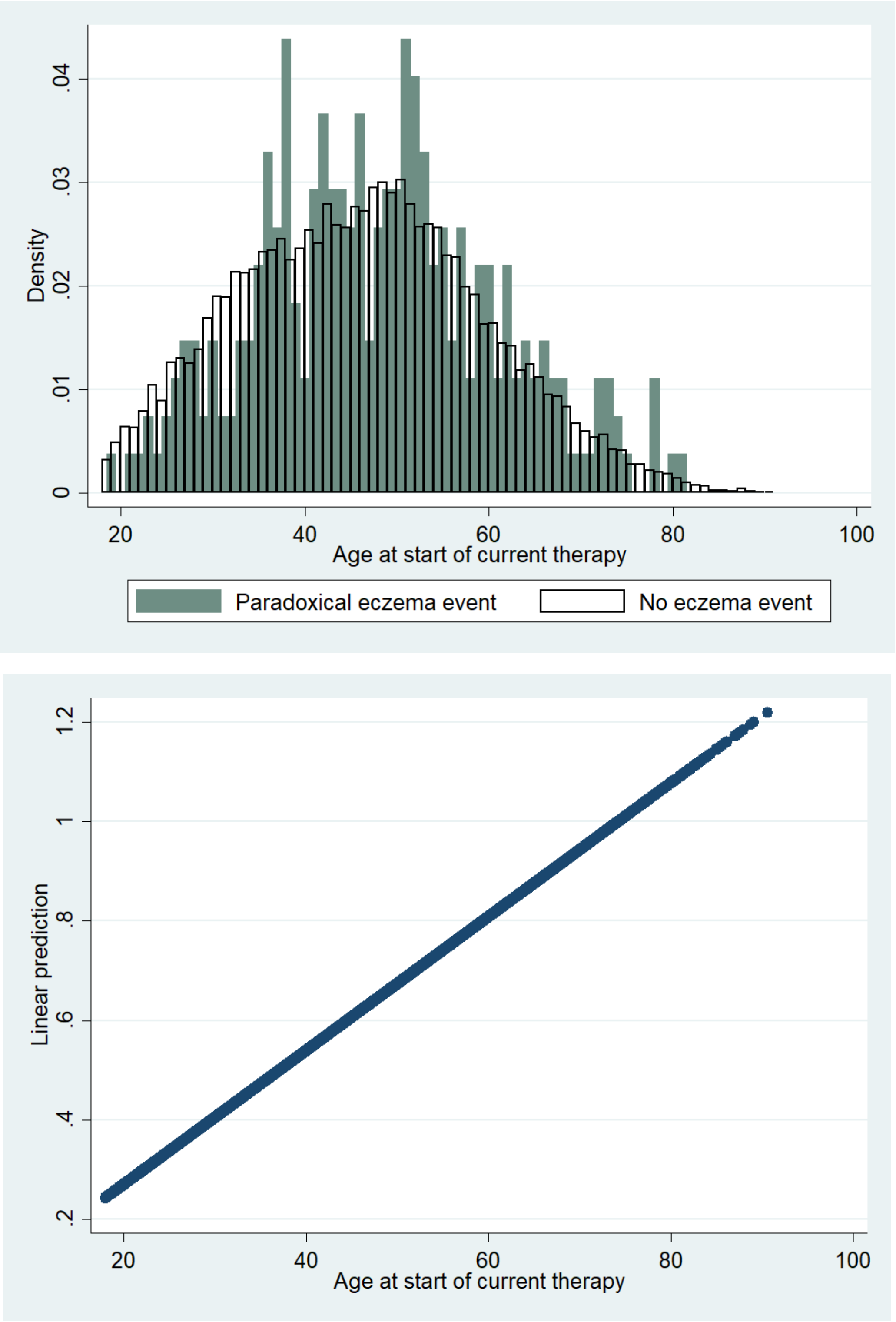

eFigure 2. Distribution of age of onset in paradoxical eczema cases.
A) Histogram of number of exposures by age at start of exposure, split by whether the exposure resulted in paradoxical eczema or not. B) Plot demonstrating linear increase between age and the survival analysis linear predictor.

© 2023 Al-Janabi A et al. *JAMA Dermatology.*

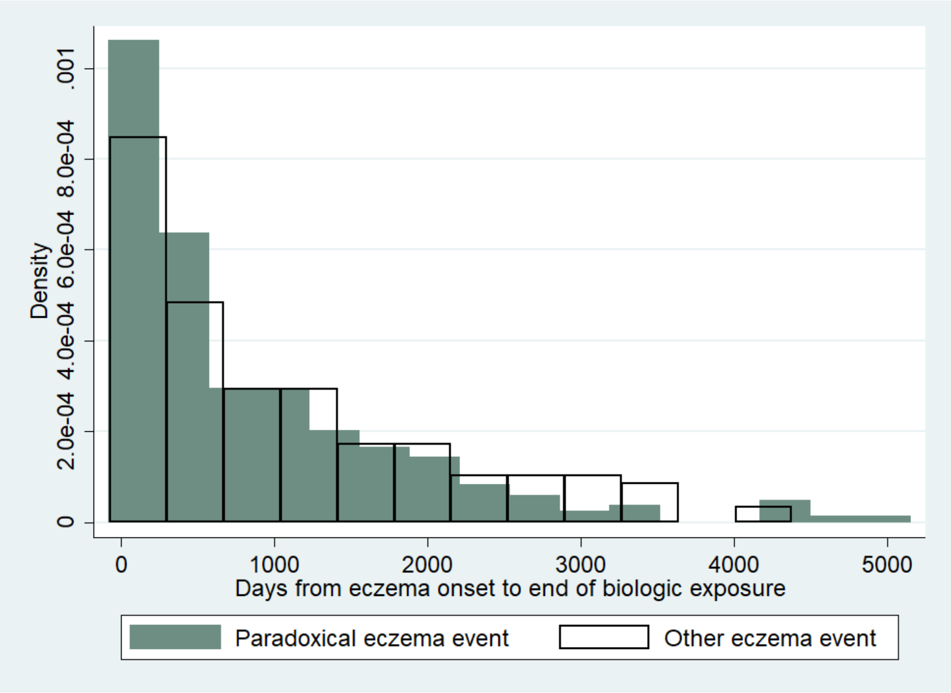

eFigure 3. Days to biologic discontinuation following onset of paradoxical eczema or other eczema phenotypes.

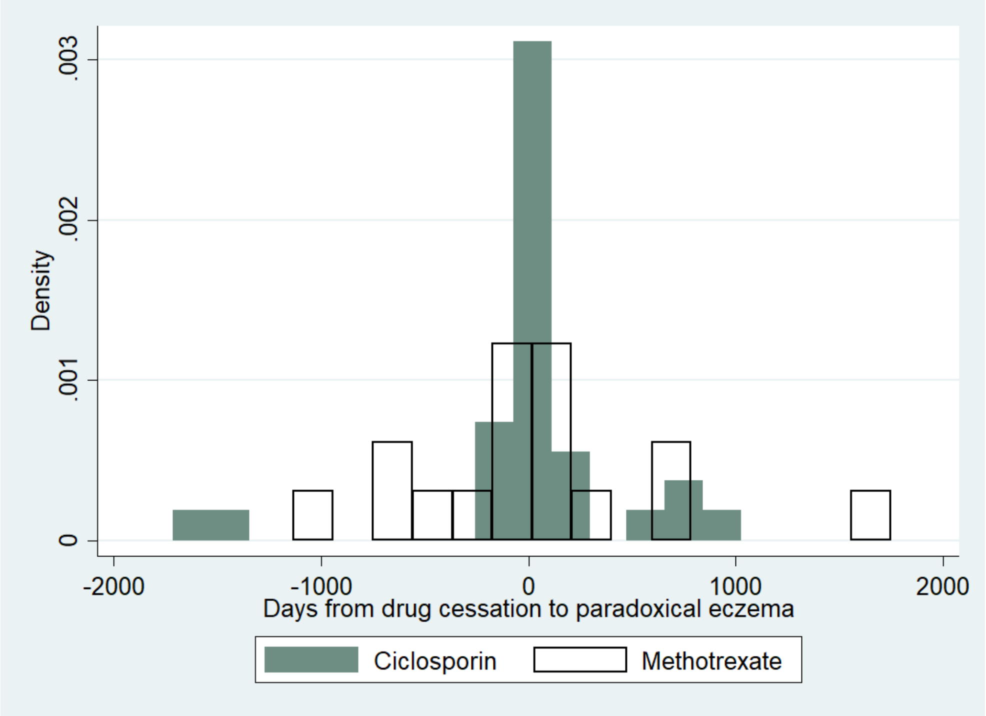

eFigure 4. Histogram showing days from cessation of methotrexate or ciclosporin, taken concurrently with a biologic at biologic initiation, to onset of paradoxical eczema.

© 2023 Al-Janabi A et al. *JAMA Dermatology.*

eTable 1. Confounder bias before and after inverse probability treatment weighting by propensity score.

	IL-17i		IL-12/23i		IL-23i	
	**Before weighting (%)**	**After weighting (%)**	**Before weighting (%)**	**After weighting (%)**	**Before weighting (%)**	**After weighting (%)**
Age	0.154	0.005	0.064	0.002	0.233	-0.008
Male sex	0.007	0.006	0.014	0.006	-0.003	0.000
Ethnicity						
Black	0.032	0.002	0.018	-0.002	0.041	0.004
Chinese	0.016	0.000	0.003	0.001	-0.001	0.000
Other[a]	0.024	0.001	0.012	0.001	0.007	-0.002
South Asian	0.082	0.002	0.048	-0.003	0.108	-0.004
White	-0.091	-0.002	-0.050	0.002	-0.103	0.003
Psoriatic arthritis	0.080	0.001	-0.173	-0.001	-0.177	-0.001
Atopic dermatitis	0.030	0.000	0.015	-0.002	0.060	0.003
Asthma	0.009	-0.002	0.009	0.002	0.082	-0.007
Hay fever	-0.005	-0.001	-0.024	-0.002	0.037	-0.001
Erythrodermic psoriasis	-0.009	0.001	0.011	0.003	-0.026	-0.001
Generalised pustular psoriasis	0.022	0.003	-0.001	-0.002	-0.024	-0.010
Palmoplantar pustulosis	-0.003	0.002	-0.027	0.003	-0.004	-0.001

Data displayed as standardised mean differences for biologic classes relative to tumour necrosis factor inhibitors.
IL, interleukin; IL-17i, IL-17 inhibitor; IL-12/23i, IL-12/23 inhibitor; IL-23i, IL-23 inhibitor.
[a] "Other" ethnicities were either defined as other by the study participant, or were missing (n=20).

eTable 2. Missing data from observations included in primary analysis (n=24,997).

Variable	Missing data, n(%)
Biologic exposure	0 (0)
Age	0 (0)
Sex	0 (0)
Ethnicity	20 (0.08)
Psoriatic arthritis	0 (0)
Atopic dermatitis	0 (0)
Asthma	0 (0)
Hay fever	0 (0)
Erythrodermic psoriasis	16 (0.06)
Generalised pustular psoriasis	37 (0.15)
Palmoplantar pustulosis	54 (0.22)

© 2023 Al-Janabi A et al. *JAMA Dermatology.*

eTable 3. Missing data from observations included in first-line biologic sensitivity analysis (n=11,732).

Variable	Missing data, n(%)
Biologic exposure	0 (0)
Age	0 (0)
Sex	0 (0)
Ethnicity	4 (0.03)
Psoriatic arthritis	0 (0)
Atopic dermatitis	0 (0)
Asthma	0 (0)
Hay fever	0 (0)
Erythrodermic psoriasis	8 (0.07)
Generalised pustular psoriasis	18 (0.15)
Palmoplantar pustulosis	23 (0.20)
Smoking	930 (7.93)
Alcohol	996 (8.49)

eTable 4. Features of paradoxical eczema events (n=265).

Clinical feature	N	%
**Site**		
Not specified	61	23
Generalised	16	6
Scalp	4	1.5
Face or neck	68	26
Trunk	35	13
Flexures	27	10
Limbs not otherwise specified	61	23
Hands or feet	33	13
Ears	5	2
Genitals	7	3
**Signs and symptoms**		
Pruritus	49	19
Pain	9	3
Redness	18	7
Swelling	8	3
Dry	11	4
Vesicles or blisters	6	2
Lichenification	5	2
Fissure	5	2
**Investigations**		
Biopsy shows eczema/spongiosis	20	NA
Biopsy shows eczema & psoriasis features	1	NA
Eosinophilia	1	NA
Elevated IgE	1	NA
**Treatments**		
Not specified	108	41
Topical	115	44
Pause biologic	3	1
Stop biologic	5	2
Switch biologic	12	5
Phototherapy	5	2
Prednisolone	12	5
Methotrexate	3	1

© 2023 Al-Janabi A et al. *JAMA Dermatology.*

Ciclosporin	2	1
Oral antibiotics	20	8
Antihistamines	8	3
Oral retinoid	2	1
Admitted to hospital	4	2

IgE, immunoglobulin E; NA, not applicable.

eTable 5. Characteristics of participants with one versus more than one paradoxical eczema event (index event only).

	Single event group (n=221)	Multiple event group (n=20)
**Age, median (IQR)**	49 (39-57)	46 (36-53)
**Sex, n(%)**		
Female	118 (53)	11 (55)
Male	103 (47)	9 (45)
**Ethnicity, n(%)**		
Black	2 (1)	0 (0)
Chinese	3 (1)	1 (5)
Other[a]	6 (3)	0 (0)
South Asian	11 (5)	3 (15)
White	199 (90)	16 (80)
**Atopy at baseline, n(%)**		
AD	17 (8)	2 (10)
Asthma	30 (14)	2 (10)
Hay fever	3 (1)	4 (20)
**PsA at baseline, n(%)**	67 (30)	8 (40)
**Other psoriasis phenotypes, n(%)**		
Erythrodermic	39 (18)	4 (20)
Generalised pustular	10 (5)	1 (5)
Palmoplantar pustulosis	7 (3)	0 (0)
**Biologic class, n(%)[b]**		
TNFi	111 (50)	16 (80)
IL-17i	41 (19)	2 (10)
IL-12/23i	63 (28)	2 (10)
IL-23i	6 (3)	0 (0)
**Combined with non-biologic systemic at biologic initiation, n(%)[b]**		
Methotrexate	14 (6)	2 (10)
Acitretin	1 (0)	0 (0)
Hydroxycarbamide	0 (0)	0 (0)
Apremilast	1 (0)	0 (0)
Mycophenolate mofetil	0 (0)	0 (0)
Ciclosporin	18 (8)	5 (25)
Dimethyl fumarate	2 (1)	1 (5)
**Combined with non-biologic systemic at any point, n(%)[b]**		
Methotrexate	27 (12)	3 (15)
Acitretin	5 (2)	0 (0)
Hydroxycarbamide	1 (0)	0 (0)
Apremilast	3 (1)	0 (0)
Mycophenolate mofetil	0 (0)	0 (0)
Ciclosporin	27 (12)	7 (35)
Dimethyl fumarate	4 (2)	1 (5)

[a] "Other" ethnicities were either defined as other by the study participant, or were missing (n=20).
[b]Drug-related statistics for both groups reflect those at the time of initiation of the biologic associated with the index paradoxical eczema event.

eTable 6. Propensity weight-adjusted Cox proportional hazards model of paradoxical eczema by biologic drug, using guselkumab as the reference category.

Biologic	Hazard ratio	95% CI	P-value
Guselkumab (reference)			
Adalimumab	3.62	1.40-9.32	0.008
Certolizumab pegol	4.90	1.12-21.42	0.04
Etanercept	3.47	1.22-9.87	0.02
Infliximab	3.44	1.00-11.81	0.05
Brodalumab	4.89	1.35-17.68	0.02
Ixekizumab	4.04	1.35-12.07	0.01
Secukinumab	3.53	1.33-9.35	0.01
Bimekizumab[a]	NA	NA	NA
Ustekinumab	3.14	1.20-8.22	0.02
Risankizumab	2.83	0.69-11.59	0.15
Tildrakizumab[a]	NA	NA	NA

CI, confidence interval.
[a]The data for bimekizumab and tildrakizumab are not shown due to unstable effect estimates, resulting from the low number of exposures and absence of paradoxical eczema events attributed to these drugs.

eTable 7. Propensity weight-adjusted Cox proportional hazards model of paradoxical eczema by biologic class or biologic drug, without an exposure risk window (sensitivity analysis).

	Hazard ratio	95% CI	P-value
Model 1 - biologic class (TNFi reference category)			
IL-17i	1.05	0.75-1.47	0.77
IL-12/23i	0.92	0.69-1.24	0.59
IL-23i	0.41	0.19-0.88	0.02
Model 2 - individual biologics (adalimumab reference category)			
Certolizumab pegol	1.54	0.48-4.90	0.47
Etanercept	0.85	0.50-1.46	0.56
Infliximab	0.71	0.26-1.93	0.50
Brodalumab	1.48	0.60-3.68	0.39
Ixekizumab	1.13	0.60-2.13	0.70
Secukinumab	0.95	0.65-1.40	0.79
Bimekizumab[a]	NA	NA	NA
Ustekinumab	0.90	0.66-1.21	0.48
Guselkumab	0.24	0.084-0.71	0.010
Risankizumab	0.89	0.31-2.59	0.83
Tildrakizumab[a]	NA	NA	NA

CI, confidence interval; IL, interleukin; IL-17i, IL-17 inhibitor; IL-12/23i, IL-12/23 inhibitor; IL-23i, IL-23 inhibitor; TNFi, tumour necrosis factor inhibitor.
[a]The data for bimekizumab and tildrakizumab are not shown due to unstable effect estimates, resulting from the low number of exposures and absence of paradoxical eczema events attributed to these drugs.

© 2023 Al-Janabi A et al. *JAMA Dermatology.*

eTable 8. Propensity weight-adjusted Cox proportional hazards model of paradoxical eczema by biologic class or biologic drug, limited to first-line therapy only (sensitivity analysis).

	Hazard ratio	95% CI	P-value
Model 1 - biologic class (TNFi reference category)			
IL-17i	1.07	0.67-1.71	0.77
IL-12/23i	0.84	0.57-1.25	0.39
IL-23i	0.17	0.023-1.19	0.07
Model 2 - individual biologics (adalimumab reference category)			
Certolizumab pegol	5.41	1.66-17.57	0.005
Etanercept	0.45	0.22-0.95	0.04
Infliximab	0.85	0.21-3.46	0.82
Brodalumab	1.61	0.40-6.55	0.50
Ixekizumab	1.17	0.42-3.23	0.76
Secukinumab	0.91	0.53-1.55	0.72
Bimekizumab[a]	NA	NA	NA
Ustekinumab	0.77	0.51-1.15	0.20
Guselkumab	0.21	0.030-1.55	0.12
Risankizumab[a]	NA	NA	NA
Tildrakizumab[a]	NA	NA	NA
Model 3 - smoking and alcohol[b]			
Smoking			
Previous smoker	0.89	0.60-1.32	0.56
Current smoker	0.79	0.50-1.25	0.31
Currently drinks alcohol	1.10	0.75-1.60	0.64

CI, confidence interval; IL, interleukin; IL-17i, IL-17 inhibitor; IL-12/23i, IL-12/23 inhibitor; IL-23i, IL-23 inhibitor; NA, not applicable; TNFi, tumour necrosis factor inhibitor.

[a] The data for bimekizumab, risankizumab and tildrakizumab are not shown due to unstable effect estimates, resulting from the low number of exposures and absence of paradoxical eczema events attributed to these drugs.

[b] Model 3 included biologic class, smoking status and alcohol consumption status as covariates. Smoking status consisted of three categories, with never smokers as the reference group. Alcohol consumption was a binary variable indicating whether the participant was consuming alcohol at baseline or not.

eTable 9. Number of exposures linked to eczema or dermatitis adverse events other than paradoxical eczema.

Adverse event phenotype	N
Asteatotic eczema	4
Chronic actinic dermatitis	1
Contact dermatitis	46
Discoid eczema	3
Hand or foot eczema (unspecified cause)	11
Photosensitive eczema	1
Pompholyx eczema	16
Seborrheic dermatitis	38
Stasis/venous eczema	39
Total[a]	159

[a] Four adverse event descriptions were consistent with dual phenotypes: one with contact and seborrheic dermatitis, and three with pompholyx and paradoxical eczema. For the sensitivity analyses, any paradoxical eczema events which were included in the primary analysis were excluded.

© 2023 Al-Janabi A et al. *JAMA Dermatology.*

eTable 10. Propensity weight-adjusted Cox proportional hazards model for risk of other eczema phenotypes by biologic class, biologic drug or other covariates (sensitivity analysis).

	Hazard ratio	95% CI	P-value
Model 1 - biologic class (TNFi reference category)			
IL-17i	1.14	0.72-1.81	0.57
IL-12/23i	1.07	0.74-1.56	0.77
IL-23i	1.25	0.65-2.44	0.50
Model 2 - individual biologics (adalimumab reference category)			
Certolizumab pegol	1.08	0.15-7.79	0.94
Etanercept	1.26	0.69-2.31	0.69
Infliximab	0.94	0.29-3.00	0.91
Brodalumab	0.75	0.10-5.42	0.77
Ixekizumab	0.64	0.19-2.12	0.47
Secukinumab	1.37	0.84-2.26	0.21
Bimekizumab[a]	NA	NA	NA
Ustekinumab	1.12	0.75-1.66	0.58
Guselkumab	1.25	0.55-2.83	0.59
Risankizumab	1.82	0.64-5.19	0.26
Tildrakizumab[a]	NA	NA	NA
Model 3 - other baseline clinical variables[b]			
Age	1.02	1.01-1.04	<0.001
Male	0.78	0.56-1.09	0.14
Ethnicity			
Black	1.29	0.17-9.69	0.80
Chinese	5.74e-17	3.89e-17 - 8.46e-17	<0.001
Other[c]	0.61	0.15-2.51	0.50
South Asian	0.85	0.39-1.88	0.69
Atopic dermatitis	2.95	0.93-9.43	0.07
Asthma	1.38	0.87-2.19	0.17
Hay fever	4.10	1.54-10.90	0.005
Psoriatic arthritis	1.13	0.79-1.61	0.50
Erythrodermic psoriasis	0.69	0.41-1.13	0.14
Generalised pustular psoriasis	1.17	0.56-2.48	0.67
Palmoplantar pustulosis	0.58	0.15-2.32	0.44

CI, confidence interval; IL, interleukin; IL-17i, IL-17 inhibitor; IL-12/23i, IL-12/23 inhibitor; IL-23i, IL-23 inhibitor; TNFi, tumour necrosis factor inhibitor.
[a]The data for bimekizumab and tildrakizumab are not shown due to unstable effect estimates, resulting from the low number of exposures and absence of eczema events attributed to these drugs.
[b]The Cox-proportional hazards model for model 3 included clinical covariates in addition to biologic class.
[c] “Other” ethnicities were either defined as other by the study participant, or were missing (n=20).

© 2023 Al-Janabi A et al. *JAMA Dermatology.*

eTable 11. Propensity weight-adjusted Cox proportional hazards model for risk of paradoxical eczema by biologic class or other covariates, adjusting time-varying use of non-biologic systemic therapies (sensitivity analysis).

	Hazard ratio	95% CI	P-value
Biologic class (TNFi reference)			
IL-17i	1.06	0.76-1.46	0.74
IL-12/23i	0.87	0.65-1.16	0.33
IL-23i	0.40	0.19-0.82	0.013
Concomitant non-biologic[a]			
Methotrexate	0.62	0.37-1.04	0.07
Ciclosporin	3.28	2.03-5.30	<0.001
Acitretin	0.36	0.05-2.61	0.31
Apremilast	1.05	0.15-7.50	0.96
Dimethyl fumarate	2.47	0.69-8.79	0.16
Age	1.02	1.01-1.03	<0.001
Male	0.62	0.47-0.82	<0.001
Ethnicity			
Black	1.36	0.32-5.76	0.67
Chinese	2.69	0.98-7.41	0.0656
Other[b]	1.11	0.48-2.55	0.81
South Asian	1.30	0.69-2.46	0.42
Atopic dermatitis	12.41	6.92-22.26	<0.001
Asthma	0.94	0.58-1.52	0.79
Hay fever	3.53	1.37-9.09	<0.001
Psoriatic arthritis	1.25	0.93-1.69	0.14
Erythrodermic psoriasis	1.11	0.77-1.60	0.58
Generalised pustular psoriasis	0.84	0.44-1.60	0.59
Palmoplantar pustulosis	1.10	0.51-2.38	0.80

CI, confidence interval; IL, interleukin; IL-17i, IL-17 inhibitor; IL-12/23i, IL-12/23 inhibitor; IL-23i, IL-23 inhibitor; TNFi, tumour necrosis factor inhibitor.

[a]Concomitant non-biologic included as a categorical variable. Hydroxycarbamide and mycophenolate were excluded due to low sample numbers.

[b]"Other" ethnicities were either defined as other by the study participant, or were missing (n=20).

References

1. Al-Janabi A, Foulkes AC, Mason K, Smith CH, Griffiths CEM, Warren RB. Phenotypic switch to eczema in patients receiving biologics for plaque psoriasis: a systematic review. J Eur Acad Dermatol Venereol. 2020;34(7):1440-8.

© 2023 Al-Janabi A et al. *JAMA Dermatology.*
