# Supplementary material for: Risk of Paradoxical Eczema in Patients Receiving Biologics for Psoriasis
Source: JAMA Dermatol. 2023 Dec 6;160(1):71–9. doi: 10.1001/jamadermatol.2023.4846 (PMC10701661; doi:10.1001/jamadermatol.2023.4846)
Supplement: Supplement 2. — BADBIR Study Group [file jamadermatol-e234846-s002.pdf]

Supplemental Online Content: Nonauthor Collaborators
*First name, last name, and suffix (if applicable) are required and will appear in PubMed.

***Group Name(s): BADBIR Study Group**

***First Name and Middle Initial(s)**	***Last Name**	***Suffix (eg, Jr, III)**	Academic Degrees	Institution	Location (city, state/province, country)	Role or Contribution, eg, chair, principal investigator	Group (if more than 1 Group listed in the byline) and/or Subgroup (eg, Steering Committee)
Philip	Laws						
Shehnaz	Ahmed						
Jonathan	Barker						
Anthoney	Bewley						
Ian	Evans						
Philip	Hampton						
Olivia	Hughes						
Brian	Kirby						
Elise	Kleyn						
Mark	Lunt						
Teena	Mackenzie						
Kathy	McElhone						
Tess	McPherson						
Simon	Morrison						
Alexa	Shipman						
Shernaz	Walton						
Christina	Ye						
Anja	Strangfeld						
Girish	Gupta						
Richard	Weller						
Vera	Zietemann						
