# Supplementary material for: Risk of Paradoxical Eczema in Patients Receiving Biologics for Psoriasis
Source: JAMA Dermatol. 2023 Dec 6;160(1):71–9. doi: 10.1001/jamadermatol.2023.4846 (PMC10701661; doi:10.1001/jamadermatol.2023.4846)
Supplement: Supplement 3. — Data Sharing Statement [file jamadermatol-e234846-s003.pdf]

Data Sharing Statement

Al-Janabi. Risk of Paradoxical Eczema in Patients Receiving Biologics for Psoriasis. *JAMA Dermatol*. Published December 06, 2023. doi:10.1001/jamadermatol.2023.4846

Data
Data available: No

Additional Information
Explanation for why data not available: Restrictions apply to the availability of these data due to patient consent and licencing agreements; data were used under license for this study. The authors therefore cannot make these data publicly available. In order to access the data set, please see the process to apply: http://www.badbir.org/Publications/DataAccess/
